# Liver X Receptor Beta Regulates Glial Dynamics and Cortical Network Remodeling in a Freezing Lesion–Cortical Dysplasia Model

**DOI:** 10.1111/cns.70671

**Published:** 2025-11-26

**Authors:** Zhi Zhang, Di Du, Min Song, Jie Li, Xinning Dong, Yiwen Mei, Jinwei Zhang, Ming Zhang, Yuan Ma, Sixun Yu, Haifeng Shu, Xin Chen

**Affiliations:** ^1^ Department of Neurosurgery General Hospital of Western Theater Command Chengdu China; ^2^ Department of Hepatobilialy Surgery General Surgery Center, General Hospital of Western Theater Command Chengdu China; ^3^ Department of Anesthesiology Chongqing University Fuling Hospital Chongqing China; ^4^ Department of Neurosurgery Sichuan Tianfu New Area People's Hospital Chengdu Sichuan Province China; ^5^ Emergency Department The 945th Hospital of the Joint Logistics Support Force of the Chinese People's Liberation Army Ya'an China

**Keywords:** brain lipid‐binding protein, cortical network remodeling, focal cortical dysplasia, glial function, liver X receptor beta

## Abstract

**Background:**

Focal cortical dysplasia (FCD) is a leading cause of drug‐resistant epilepsy, characterized by cortical malformations and aberrant neuronal‐glial interactions. Recently, the role of Liver X Receptor Beta (LXRβ) in neurodevelopment has attracted considerable attention, although its involvement in FCD pathogenesis remains unclear.

**Methods:**

We established a freezing lesion‐cortical dysplasia (FL‐CD) model in neonatal mice to mimic the pathological features of FCD. We evaluated the expression of LXRβ and its downstream target, brain lipid‐binding protein (BLBP), using immunohistochemistry and Western blot analysis. LXRβ activation and inhibition were pharmacologically modulated to assess their effects on glial migration, differentiation and cortical electrophysiology. Electroencephalogram (EEG) recordings were analyzed for power spectral density and functional connectivity to further investigate alterations in cortical network activity.

**Results:**

LXRβ and BLBP were significantly downregulated in the lesion cortex during early developmental stages. Activation of LXRβ reduced gliosis, promoted astrocytic differentiation, and modified cortical oscillatory activity, as evidenced by enhanced α power and gamma band functional connectivity, along with adjustments in the theta/beta ratio. In contrast, inhibition of LXRβ exacerbated gliosis and disrupted cortical network synchronization.

**Conclusion:**

Our findings demonstrate that LXRβ plays a critical role in regulating glial migration, differentiation and cortical network remodeling in the FL‐CD model. Pharmacological modulation of LXRβ may offer a novel therapeutic strategy for restoring neural circuit stability in FCD, highlighting its potential as a molecular target for intervention in drug‐resistant epilepsy.

## Graphical abstract

LXRβ activation reduces gliosis, promotes astrocytic differentiation, and enhances cortical network synchronization in fetal cortical dysplasia, while its inhibition exacerbates disruptions. Pharmacological targeting of LXRβ emerges as a therapeutic strategy to restore neural circuit stability in drug‐resistant epilepsy.

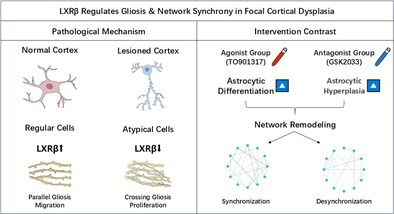

## 1 Introduction

Focal Cortical Dysplasia (FCD) is a brain developmental disease caused by abnormal neuronal proliferation, migration and differentiation [1]. It is a leading cause of drug‐resistant epilepsy, particularly in childhood, where its prevalence in focal epilepsy ranges from 5% to 25% [2]. Since epilepsy in FCD patients does not respond well to antiepileptic drugs, conventional drug treatment has a limited effect [3, 4]. Although surgery is a common treatment option, 35% of patients will experience epileptic seizures again after surgery [5]. Therefore, in order to gain a deeper understanding of the pathogenesis of FCD, it is particularly important to establish an animal model that can accurately simulate its pathological characteristics. The freezing lesion‐cortical dysplasia (FL‐CD) model effectively mimics the abnormal cortical layering and neuronal disorganization observed in FCD by inducing localized cortical damage, providing a reliable platform to study its molecular and cellular mechanisms [6].

Glial cells, especially radial glial cells (RGCs) and astrocytes, play a key role in the development and homeostasis of the cerebral cortex. Research indicates that glial dysfunction significantly affects neuronal migration and differentiation, directly contributing to the pathological progression of FCD. RGCs act as scaffolds for neuronal migration in the early stages of cortical development and differentiate into a variety of neuronal/glial lineage cells including astrocytes after neuronal migration is completed [7]. In FCD, RGCs often display abnormal persistence or impaired trans‐differentiation, leading to the formation of dysplastic neurons or ‘balloon cells’ that drive FCD pathogenesis [8]. In addition, astrocytes differentiated from RGCs may also be impaired in maintaining synaptic homeostasis, clearing glutamate, and buffering potassium ions, thereby triggering abnormal discharges in local brain tissue and increasing susceptibility to epileptic seizures [9, 10]. Therefore, both the abnormal proliferation and differentiation of RGCs and the dysregulation of astrocyte function may aggravate the malformation of cortical structure and promote the appearance of epileptic phenotypes.

Liver X Receptor Beta (LXRβ) was initially identified for its role in regulating cholesterol, lipid, and glucose metabolism [11]. Recent studies, however, highlight its significant functions in the central nervous system [12, 13]. LXRβ not only regulates the activation state of astrocytes and microglia, but is also closely related to the function of RGCs. For example, the loss of LXRβ can weaken the scaffolding function of RGCs and destroy the hierarchical structure of neurons, thereby significantly interfering with the migration of neocortical neurons [14]. Additionally, LXRβ influences the structure and differentiation of RGCs by regulating the expression of Brain Lipid‐Binding Protein (BLBP), thereby affecting neuronal migration and cortical stratification [12]. Abnormalities of LXRβ and BLBP at the molecular or functional level may play a key role in the occurrence and development of developmental cortical abnormalities [14].

Our previous studies have found that LXRβ shows specific expression and distribution in clinical lesion specimens surgically removed from FCD patients [12]. However, its specific role and potential mechanism in the pathological process of FCD have not been systematically studied. Hence, this study aims to establish and validate the FL‐CD animal model, deeply evaluate the expression dynamics of LXRβ in the pathological process of FL‐CD, and explore its possible regulatory role in glial cell function and cortical structural abnormalities, to uncover novel molecular insights into the pathogenesis of FCD.

## 2 Materials and Methods

### 2.1 Animals

In this experiment, 8–10‐week‐old SPF wild‐type C57BL/6 mice were provided by Chengdu Dashuo Animal Experimental Center and Chengdu Lilai Animal Experimental Center. The breeding method was 1 male and 3 females in the same cage. The housing conditions included: ambient temperature 22°C–26°C, relative humidity 40%–70%, a twelve‐hour light–dark cycle, free access to water and food. Pregnant female mice were housed individually, and newborn mice (P0–P1) were randomly divided into groups within 24 h after birth and received freezing injury (FL‐CD group) or sham surgery (sham group). All experimental operations and procedures followed the international animal care guidelines set forth by the Declaration of Helsinki and were approved by the Animal Care and Use Committee of the General Hospital of the Western Theater Command.

### 2.2 Establishment of the Freeze‐Lesion Model and Drug Intervention

Referring to the previous research method of our team [15], freeze‐lesioning was performed on neonatal mice (P0) to induce the formation of abnormal microgyri. The specific experimental steps are as follows: The newborn mice were placed in ice water for deep hypothermia anesthesia for about 2 min. Subsequently, the mice were fixed under a dissecting microscope, and a liquid nitrogen‐cooled copper column with a diameter of 1.5 mm was placed on the surface of the right skull at a distance from the midline for a contact time of 5–8 s. To construct the longitudinal freeze‐lesioning area, an L‐shaped copper needle was used to generate microgyri about 3 mm long in the head and tail direction on the skull. The sham group used the same operation process, but no cooling treatment was performed. After the operation, the mice were returned to the mother mouse cage for routine feeding and were given drug intervention according to the experimental design. They were divided into the following groups: (1) FL‐CD + DMSO group: DMSO was injected intraperitoneally after freezing injury; (2) FL‐CD + TO group: TO901317 (> 98%, Sigma, T2320), an LXRβ agonist, was injected intraperitoneally (TO901317 + 1% DMSO, 5 mg/mL) after freezing injury; (3) FL‐CD + GSK group: GSK2033 (> 98%, Sigma, SML1617), an LXRβ blocker, was injected intraperitoneally (GSK2033 + 1% DMSO, 5 mg/mL) after freezing injury. After the experiment, samples were collected at P2, P7, P10, and P14 to evaluate the relevant experimental indicators of each group.

### 2.3 Western Blotting

To evaluate the levels of LXRβ and BLBP proteins in the cortex of mice in the FL‐CD and sham groups, β‐actin was used as a loading control. After the brain tissues of the experimental animals were collected, they were homogenized in lysis buffer and centrifuged at 4°C. After the supernatant was collected, the protein samples were loaded on a 12% SDS‐PAGE gel for electrophoresis separation and transferred to a 0.45 μm PVDF membrane. The membrane was blocked with TBST solution containing 5% non‐fat milk at room temperature for 2 h. After blocking, the PVDF membrane was incubated with anti‐rabbit LXRβ polyclonal antibody (Abcam, Ab28479, 1:1000), anti‐BLBP antibody (Abcam, Ab32423, 1:2000) and anti‐β‐actin antibody (Boster, BA2305, 1:2000) at 4°C overnight. The next day, the PVDF membrane was washed three times with TBST for 10 min each time, and then incubated with horseradish peroxidase (HRP)‐labeled goat anti‐rabbit IgG (Servicebio, GB21303, 1:10,000) at room temperature for 1 h. Subsequently, the protein signal was detected using a chemiluminescence kit (ECL Western Blotting Substrate Kit, Merck‐Millipore, WBKLS0100) according to the manufacturer's instructions. The grayscale value of the protein band was quantitatively analyzed using ImageJ software.

### 2.4 Immunohistochemistry and Histology

The animals were anesthetized by intraperitoneal injection of 4% chloral hydrate (350 mg/kg), followed by transcardial perfusion with 20–40 mL of chilled saline and 20–40 mL of 4% paraformaldehyde (PFA). After perfusion, the brain tissue was fixed in 4% PFA overnight, and the tissue was trimmed according to the obvious area of microgyri in the right cortex. The fixed tissue was paraffin‐embedded, and the coronal sections were sliced at a thickness of 10 μm and attached to poly‐L‐lysine‐coated slides. Two sections were taken from each paraffin block for hematoxylin–eosin (HE) staining and immunohistochemistry (IHC) detection, respectively.

After dewaxing and gradient hydration, the paraffin sections were incubated in 0.3% hydrogen peroxide solution for 30 min to eliminate endogenous peroxidase activity. Antigen retrieval was completed using microwave‐heated phosphate‐buffered saline (PBS, 0.01 M, pH 7.3). The sections were blocked in 10% normal goat serum for 1 h at room temperature and then incubated with the following primary antibodies at 4°C overnight: anti‐LXRβ antibody (Abcam, Ab28479, 1:400), anti‐BLBP antibody (Abcam, Ab32423, 1:400). The next day, the sections were washed three times with PBS and incubated with horseradish peroxidase‐conjugated dextran polymer‐conjugated secondary antibody: biotin‐labeled goat anti‐rabbit IgG (ready‐to‐use) at 37°C for 1 h. Visualization of the immunoreaction was done with 3,3‐diaminobenzidine (Boster, DA1015), and the sections were counterstained with hematoxylin. Average optical density was measured using ImageJ software.

### 2.5 Immunofluorescence Staining

Mouse brain tissue was fixed by perfusion and then dehydrated in 15% and 30% sucrose solutions for 48 h to prevent tissue damage during sectioning. The brain tissue was then cut into 30 μm thick coronal sections using a sliding cryostat. The sections were incubated in blocking solution containing goat serum at room temperature for 100 min and then incubated with the following primary antibodies at 4°C for 24 h: anti‐LXRβ antibody (Abcam, Ab28479, 1:400), anti‐BLBP antibody (Abcam, Ab32423, 1:500), anti‐Iba1 antibody (GeneTex, GTX632426, 1:500), anti‐NeuN antibody (GeneTex, GTX30773, 1:500) and anti‐GFAP antibody (Abcam, Ab10062, 1:1000). After incubation, the sections were washed three times with PBS and incubated with CY3‐labeled secondary antibody (Servicebio, GB21303, 1:400) at room temperature for 1 h to achieve fluorescent labeling of protein signals. After staining, the sections were counterstained with DAPI (Servicebio, G1012). Fluorescence images were collected using a Nikon A1R confocal microscope (Nikon, Japan) and quantitatively analyzed using ImageJ software.

### 2.6 Electroencephalogram Recording and Analysis

After 8 weeks of modeling, mice were anesthetized by intraperitoneal injection of 4% chloral hydrate (350 mg/kg), and the skull surface was exposed. The experiment used a 0.05‐in. diameter silver wire electrode, whose insulation layer extended to 0.5 mm from the cut end of the electrode. Six electrodes were soldered to micro connectors, of which channels 1 and 3 were used to record the cortex on the side of the freezing injury, channels 2 and 4 were used to record the contralateral cortex, and channels 5 and 6 were used as ground electrodes and reference electrodes (Figure 6), respectively. To fix the electrodes, a small piece of Gelfoam was covered on each electrode hole and dental cement was used to fix the electrodes to the skull surface. After the surgery, the mice were returned to the cage for 1 week of recovery.

The EEG recording was completed by connecting the implanted electrodes to a digital electroencephalograph (RM6250, Schchengyi, China), and the sampling rate was set to 800 Hz. During recording, the mice first moved freely in the recording box for 10 min to adapt to the environment, and then the EEG recording started for at least 90 min continuously, during which the mice were free to move. For further analysis, 5 min of EEG data were selected from a wakeful, non‐movement resting state.

The processing of raw EEG data was performed in MATLAB (MathWorks). First, artifact detection was used to remove abnormal data segments with amplitudes exceeding ±100 μV, and data gaps were repaired by linear interpolation. Then, a 50 Hz notch filter was applied to remove power frequency interference, and a 0.1–50 Hz bandpass filter was performed to retain key EEG frequency band information. To eliminate the baseline drift effect, the data of each channel were baseline corrected by mean removal. The preprocessed data were used for quantitative analysis of power spectrum and functional connectivity.

The power spectral density of different frequency bands, including Delta (1–4 Hz), Theta (4–8 Hz), Alpha (8–13 Hz), Beta (13–30 Hz), and Gamma (30–50 Hz) bands, was calculated by fast Fourier transform. The power spectral density results were normalized to relative power to quantify the activity level of each frequency band. The ratio of the relative power of theta and beta bands (theta/beta ratio) was calculated to evaluate the dynamic frequency band balance. The coherence analysis method was used to quantify the synchronization pattern between channels and calculate the functional connectivity of different frequency bands. Based on the analysis results, a 4 × 4 functional connectivity matrix was constructed to compare the functional connectivity characteristics of different experimental groups in different frequency bands.

### 2.7 Statistics

Statistical analysis was performed using GraphPad Prism 8 software (GraphPad Software Inc.) or R (v4.1.0), and the specific statistical methods are described in detail in the legends of each figure. Experimental data are presented as mean ± standard error (mean ± SEM). The differences between two groups were analyzed using independent sample *t*‐test; for comparisons of three or more groups, one‐way analysis of variance (ANOVA) was used, combined with Tukey's multiple comparison test or Games‐Howell's multiple comparison test to assess differences between groups. The statistical significance level was set as: *p < 0.05, **p < 0.01, ***p < 0.001.

## 3 Results

### 3.1 Evaluation of the FL‐CD Model

**FIGURE 1:**
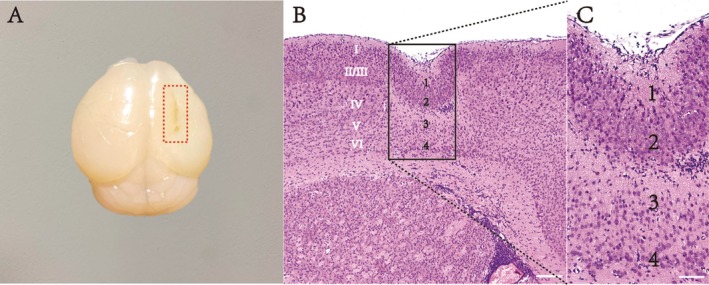
Schematic diagram of microgyria and HE staining in the right cortex of freezing lesion–cortical dysplasia model. (A) Brain tissue of a freezing lesion‐cortical dysplasia (FL‐CD) model. The red dotted circle highlights the presence of obvious microgyria. (B) HE staining of the cortex after freezing lesion reveals the formation of a heterogeneous cortex. (C) Detailed view of the heterogeneous cortex in the same cortex. Scale bars: (B) 500 μm, (C) 100 μm.

Local cortical freezing injury in mice resulted in the formation of prominent cortical microgyri (see Figure 1). Hemosiderin deposits were observed in most microgyri, with their content gradually decreasing over time. HE staining demonstrated that the FL‐CD model successfully reproduced a heterogeneous cortex, reducing the original six‐layer structure to four layers. Neurons were arranged in a disordered manner, and columnar polarity was lost. The injured cortex could be broadly divided into a depressed molecular layer and a superficial neuronal layer (layers 1 and 2), a middle layer with neuronal loss and a deep neuronal layer (layers 3 and 4) adjacent to the white matter. These pathological changes are consistent with those reported in our previous study [6, 15], confirming the successful establishment of the FL‐CD model.

### 3.2 Changes in the Expression of LXRβ and BLBP in the FL‐CD Model

**FIGURE 2:**
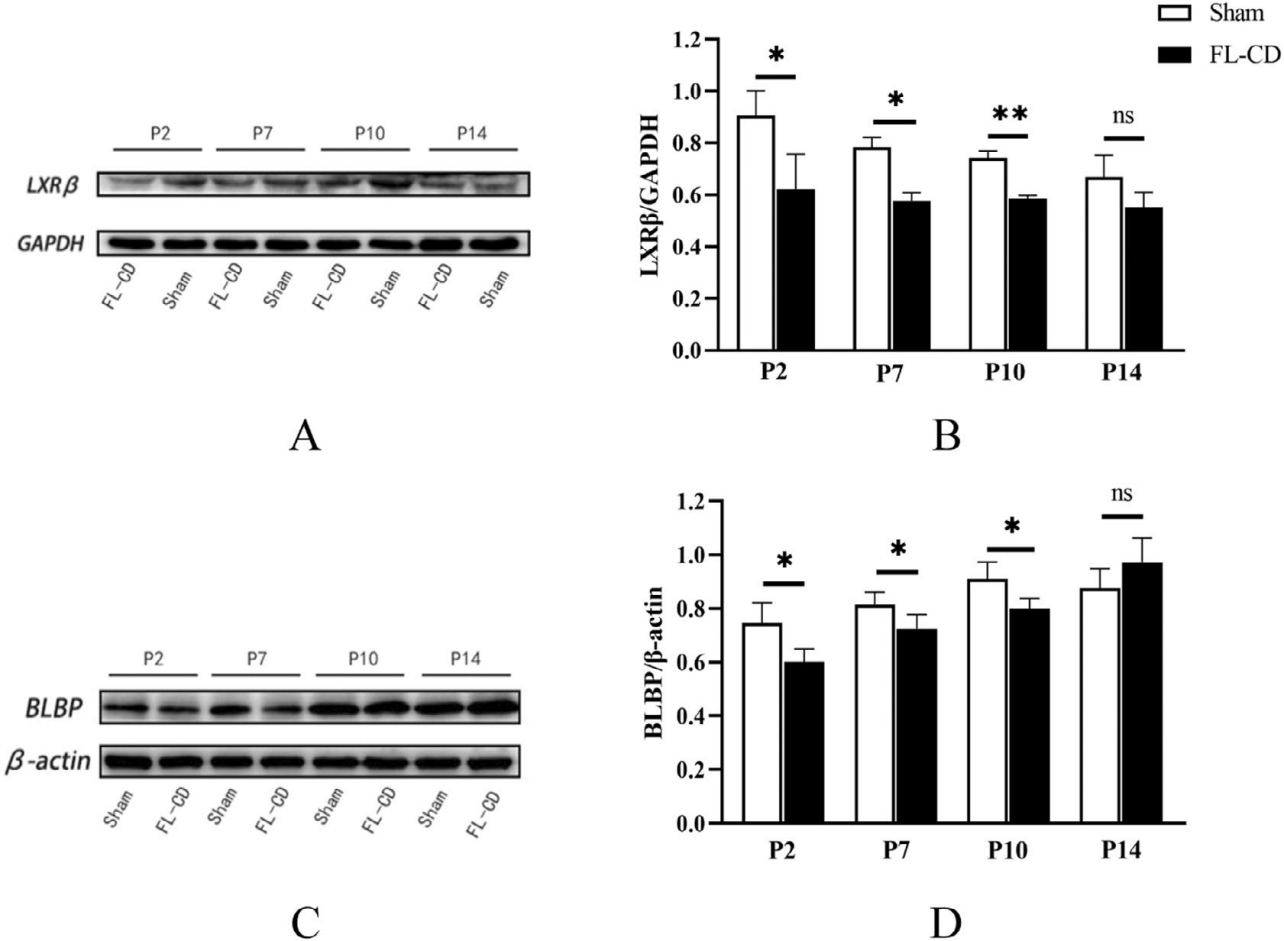
Expression levels of LXRβ and BLBP proteins in the FL‐CD and sham groups. (A) Western blot bands of LXRβ protein. (B) Relative grayscale analysis of LXRβ expression in the FL‐CD and sham group at the P2, P7, P10 and P14 time points. (C) Western blot analysis of BLBP protein; (D) Relative grayscale analysis of BLBP expression at the P2, P7, P10 and P14 time points compared to the sham group (*p < 0.05, **p < 0.01, ns, not significant). *N* = 4 in all groups.

Following the successful establishment of the model, we examined the expression changes of key molecules LXRβ and BLBP (Figure 2). As the cortex of the model mice developed, the expression of LXRβ protein gradually decreased in both the FL‐CD and sham groups. LXRβ expression in the FL‐CD group was significantly lower than in the sham group at P2, P7, and P10 (*p* < 0.01), with the most pronounced difference observed at P10 (*p* < 0.01). By P14, although LXRβ expression remained downregulated, the difference was not statistically significant (*p* > 0.05). Similarly, the RGC‐specific protein BLBP was significantly downregulated at P2, P7, and P10, but no significant difference was also observed at P14 (*p* > 0.05).

### 3.3 Immunohistochemical Localization of LXRβ and BLBP in the FL‐CD Model

**FIGURE 3:**
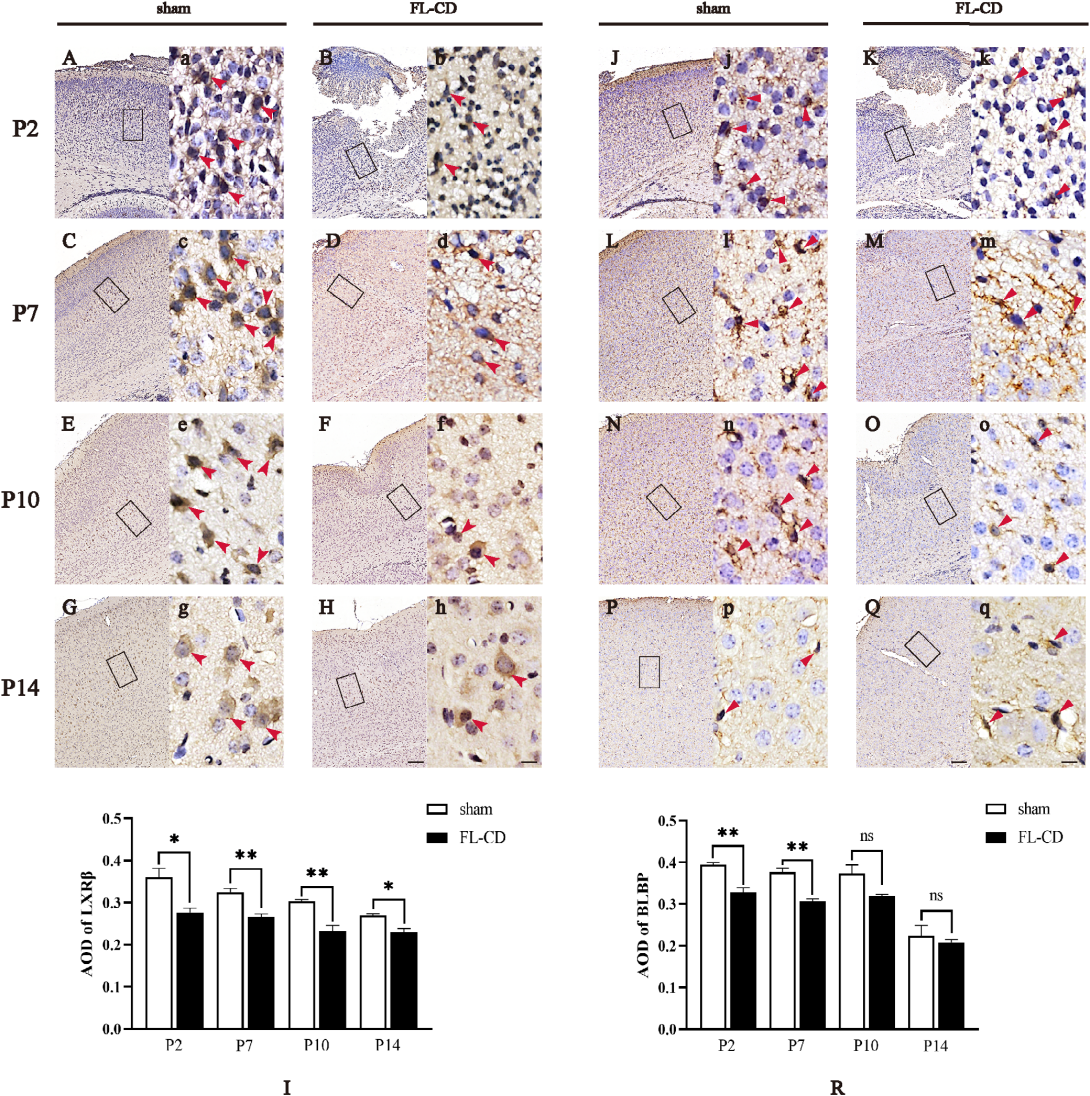
LXRβ and BLBP protein expression and localization in the cortex of FL‐CD and sham groups. (A–H) Expression and localization of LXRβ in the lesion cortex. (J–Q) Expression and localization of BLBP in the lesion cortex. (I) Statistical analysis of average optical density for LXRβ in the lesion cortex. (R) Statistical analysis of average optical density for BLBP in the lesion cortex. Scale bars: (A–Q) 100 μm, (a–q) 10 μm. *p < 0.05, **p < 0.01; ns, not significant. *N* = 3, cell densities measured from three different areas.

Given that LXRβ is a nuclear receptor associated with BLBP expression, we further assessed the expression and localization of LXRβ and BLBP in the FL‐CD and sham groups using immunohistochemical staining (see Figure 3). The FL‐CD group exhibited significant cortical damage in the early stages, characterized by extensive clusters of apoptotic cells around the lesion site and areas of cortical necrosis and detachment (Figure 3B/K). However, this phenomenon was absent in the sham group. The damaged cortex in the FL‐CD group gradually recovered, ultimately forming a characteristic ‘U’‐shaped heterogeneous cortex (Figure 3F/O). Quantitative analysis revealed that LXRβ expression in the FL‐CD group was consistently lower than in the sham group at all four time points, with the most significant differences observed at P7 and P10 (Figure 3I, *p* < 0.01). Additionally, BLBP expression in the FL‐CD group was significantly lower than in the sham group at P2 and P7 (Figure 3R, *p* < 0.01). Although BLBP expression decreased at P10, the difference did not reach statistical significance (*p* > 0.05), and no significant difference was observed at P14 (Figure 3R, *p* > 0.05).

### 3.4 Role of LXRβ and BLBP in Glial Migration and Cytological Characteristics of the FL‐CD Model

**FIGURE 4:**
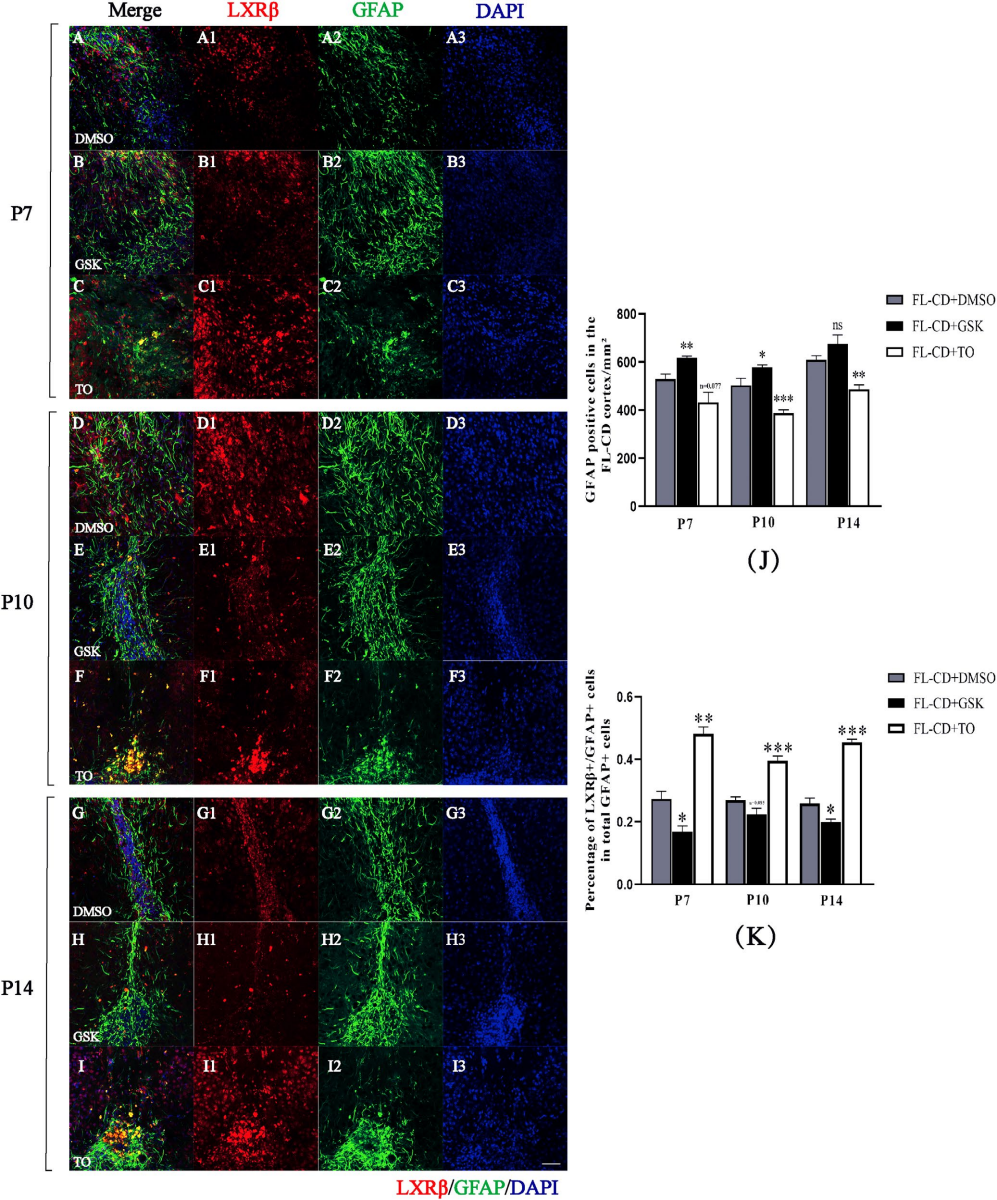
Expression and distribution of LXRβ/GFAP co‐labeled positive cells in the lesion cortex of FL‐CD model after drug intervention. (A–I) LXRβ/GFAP co‐labeling in the lesion cortex of drug‐treated FL‐CD model at different time points. (J) Statistical analysis of the number of GFAP‐positive cells in the dysplastic cortex after drug intervention; (K) Statistical analysis of the proportion of LXRβ/GFAP co‐labeled cells among total GFAP‐positive cells; DMSO: FL‐CD + DMSO, GSK: FI‐CD + GSK2033, TO: FL‐CD + TO901317. Scale bar: 100 μm. *p < 0.05, **p < 0.01, *p < 0.001, ns, not significant. *N* = 3, cell densities measured from three different areas.

**FIGURE 5:**
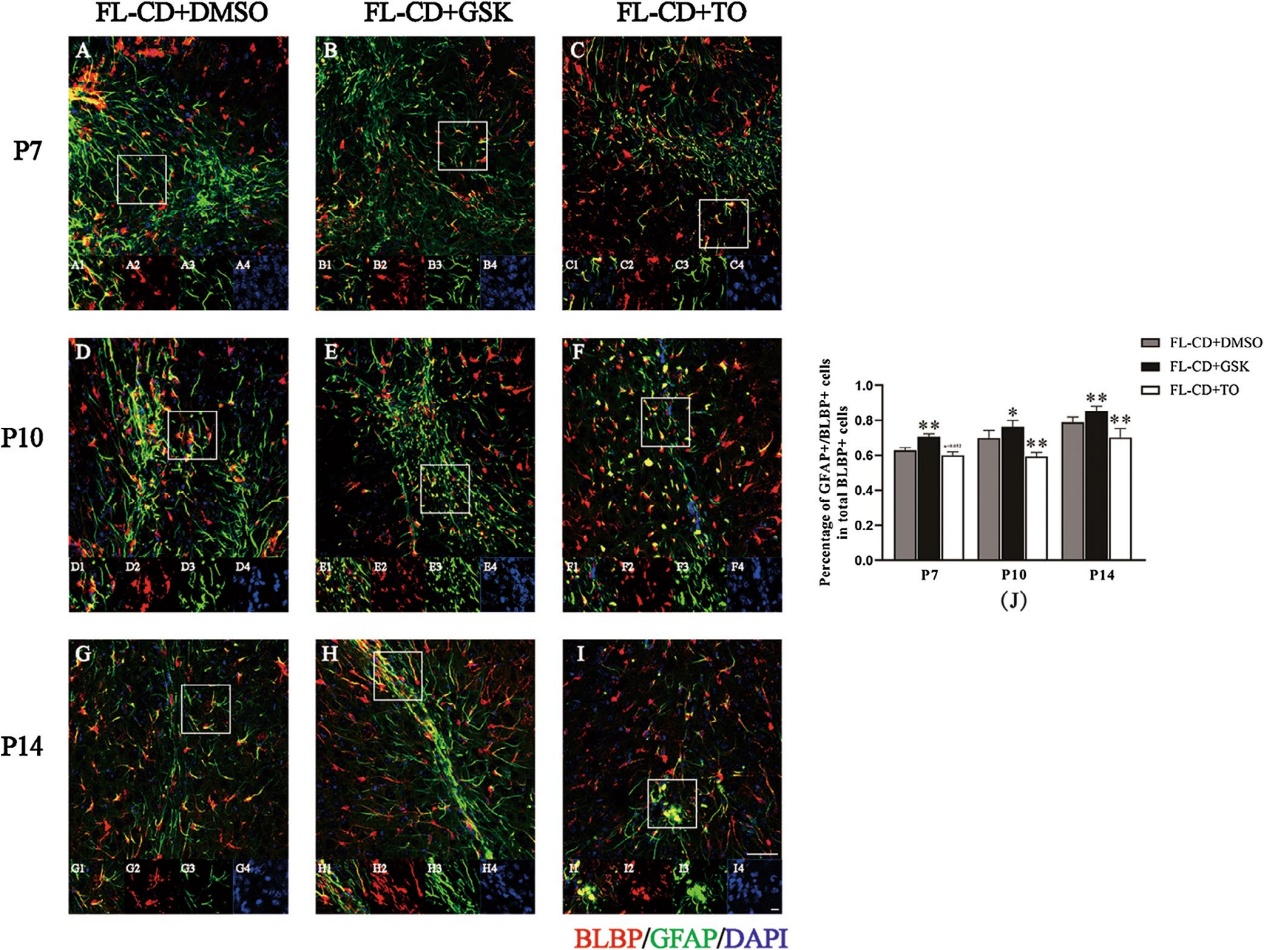
Expression and distribution of BLBP/GFAP co‐labeled positive cells in the lesion cortex of FL‐CD model after drug intervention. (A–I) BLBP/GFAP co‐labeling in the lesion cortex of drug‐treated FL‐CD model at different time points. (J) The proportion of BLBP/GFAP co‐labeled positive cells among the total number of GFAP‐positive cells. Scale bars, A–I: 50 μm, insets: 10 μm. *p < 0.05, *p* < 0.01, ns: Not significant. *N* = 3, cell densities measured from three different areas.

BLBP is a specific marker for RGCs, while GFAP specifically marks astrocytes. BLBP/GFAP co‐labeled positive cells represent the transitional stage of RGCs transforming into astrocytes. At P2, all three groups exhibited partial necrosis and structural disorder in the lesion cortex, without the formation of a typical heterogeneous cortex or “glial migration flow”. Numerous LXRβ/GFAP and BLBP/GFAP double‐labeled cells were consistently observed around the lesion site (Figure S1). Notably, some LXRβ/GFAP co‐labeled cells in the FL‐CD + GSK group exhibited typical astrocyte “branched protrusions” while most co‐labeled positive cells remained in the “cell body” state, a feature not observed in the FL‐CD + DMSO and FL‐CD + TO groups (Figure S1B). Starting from P7, the lesion cortex of the FL‐CD model gradually formed a typical heterogeneous cortex, consistent with HE staining and IHC results. All three groups displayed disordered cell arrangement, cortical thinning and significant gliosis in the lesion cortex (Figure S2). In the FL‐CD + DMSO group, the closer the “glial migration flow” was to the superficial layer, the more pronounced the gliosis (Figure S2A). In the FL‐CD + GSK group, gliosis was more significant, and the “glial migration flow” was also more evident (Figure S2B). In contrast, the FL‐CD + TO group exhibited milder cortical gliosis, and the “glial migration flow” was less apparent (Figure S2C). Similar patterns were observed at P10 and P14 (Figures 4 and 5).

To clarify the cellular localization of LXRβ and BLBP, we performed additional double‐labeling immunofluorescence with NeuN and Iba1. As shown in Figure S3, in contrast to their abundant co‐localization with GFAP‐positive astrocytes as previously described, both LXRβ and BLBP exhibited only limited overlap with NeuN‐ or Iba1‐positive cells, suggesting that their expression is largely restricted to astrocytes in the lesion area.

Further quantitative immunofluorescence analysis revealed that from P7 to P14, the number of GFAP‐positive cells in the FL‐CD + GSK group significantly increased, especially at P7 and P10 (Figure 4A–F,J). However, the number of LXRβ/GFAP co‐labeled positive cells in the FL‐CD + GSK group significantly decreased at P7 and P14, with a near‐significant difference at P10 (*p* = 0.085, Figure 4K). In the FL‐CD + TO group, the number of GFAP‐positive cells showed a reduction across all three time points with no significant difference at P7 (*p* = 0.077), but a significant decrease at P10 and P14 (Figure 4J). Concurrently, the number of LXRβ/GFAP co‐labeled positive cells significantly increased Figure 4K, primarily concentrated in the deep layers of the injured cortex (Figure 4C,F,I). At P7, P10, and P14, dense clusters of BLBP/GFAP co‐labeled positive cells were observed in the heterogeneous cortex on the lesion side (Figure 5). Quantitative analysis showed that the proportion of BLBP/GFAP co‐labeled positive cells among GFAP‐positive cells significantly increased in the FL‐CD + GSK group, while it significantly decreased in the FL‐CD + TO group at P10 and P14, with a near‐significant difference at P7 (*p* = 0.052, Figure 5J).

### 3.5 Effects of LXRβ on Cortical Function of FL‐CD Model

**FIGURE 6:**
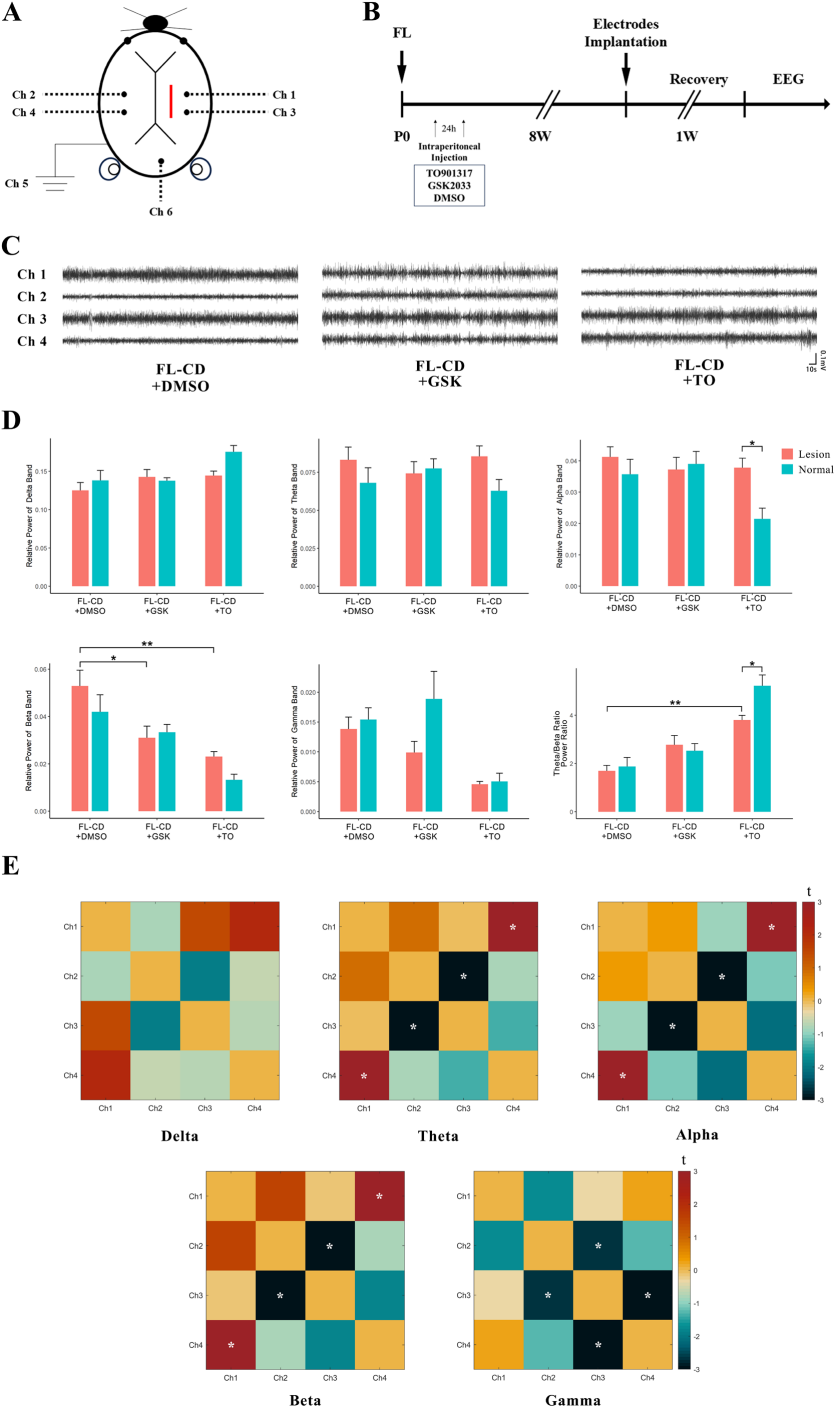
Effects of LXRβ on EEG activity and functional connectivity in FL‐CD model. (A) Schematic diagram of electrode implantation and surgical procedure. (B) Experimental procedure for drug administration and EEG recording. (C) Representative EEG traces recorded from the lesion cortex (Ch1 and Ch3) and contralateral cortex (Ch2 and Ch4) in different groups. (D) Power spectral analysis of lesion cortex and contralateral cortex in different groups. (E) Functional connectivity analysis showing coherence between different channels across various frequency bands in FL‐CD + TO group. *p < 0.05, **p < 0.01, ***p < 0.001, ns, not significant.

Figure 6A,B illustrate the schematic diagram and surgical procedure of electrode implantation. Using the implanted electrodes, we recorded EEG activity from the lesion cortex (Ch1 and Ch3) and the contralateral cortex (Ch2 and Ch4) of the FL‐CD model in different groups (Figure 6C). Relative power analysis (Figure 6D) revealed that, compared to the control group (FL‐CD + DMSO), the FL‐CD + TO group showed a significant increase in the relative power of the α band and a significant decrease in the relative power of the β band on the lesion side. In the FL‐CD + GSK group, the relative power of the β band on the injured side was significantly lower than that in the control group, while no significant difference was observed in the α band (Figure 6D). To comprehensively evaluate the dynamic balance between different frequency bands, we compared the *θ*/*β* ratio (Figure 6D). The results indicated no significant difference between the lesion and normal sides in the control group. In the FL‐CD + TO group, the *θ*/*β* ratio was significantly increased on both the normal and lesion sides, with the ratio on the normal side being significantly higher than that on the lesion side. In contrast, the *θ*/*β* ratio in the FL‐CD + GSK group increased on both sides but without significant differences (*p* > 0.05).

Additionally, functional connectivity analysis was performed to explore synchronization patterns between different brain regions across various frequency bands. The results (Figure 6E) showed that, compared to the control group, the FL‐CD + TO group exhibited significantly enhanced coherence between channels 2–3 in the Theta to Beta frequency bands and significantly reduced coherence between channels 1–4. In the Gamma frequency band, coherence between channels 2–3 and 3–4 was significantly higher in the FL‐CD + TO group than in the control group. Conversely, the FL‐CD + GSK group showed minimal changes in functional connectivity across frequency bands, with only slight decreases in coherence between some channels and no significant differences in connection strength between most brain regions compared to the control group. The overall network synchronization pattern in the inhibitor group did not exhibit significant changes similar to those observed in the agonist group (data not shown).

## 4 Discussion

This study aimed to investigate the role of LXRβ in the pathogenesis of FCD. We successfully established a FL‐CD animal model, which reproduced some of the pathological features of FCD, including cortical microgyria, disrupted cortical layering and abnormal neuronal arrangement. By detecting different developmental stages, we found that the expression of LXRβ and its downstream target BLBP was significantly downregulated in the lesion cortex, especially in the early developmental stage. Immunohistochemical analysis confirmed these reductions and highlighted associated cytopathological changes. Through pharmacological intervention with LXRβ agonists and antagonists, we found that activation of LXRβ could reduce gliosis and regulate cortical electrophysiological activity. In contrast, inhibition of LXRβ exacerbated gliosis.

In this study, the expression of LXRβ and its downstream target BLBP was significantly downregulated in the lesion cortex of the FL‐CD model, particularly during P2–P10. This phenomenon suggests that alterations in LXRβ and BLBP expression may contribute to the pathological development of FL‐CD. Previous studies have shown that LXRβ regulates lipid metabolism, neuroinflammation and cortical development during brain development [16, 17, 18]; Additionally, LXRβ has been implicated in glial cell function [19]. It is dynamically expressed during dentate gyrus development and its loss results in incomplete maturation of this hippocampal region [20]. These findings indicate that the expression of LXRβ plays a vital role in the process of neural development, and its downregulation may impair cortical development and neuronal migration. As a marker of RGCs, BLBP is involved in neuronal migration and cortical layer formation, and plays an important role in the differentiation of RGCs into astrocytes [21]. Therefore, downregulation of BLBP may disrupt the normal differentiation of RGCs into astrocytes, thereby affecting the development of the cortex. Further research indicates that reduced BLBP expression may delay the maturation of astrocytes, disrupt microenvironmental homeostasis and ultimately exacerbate cortical dysfunction and structural disorganization [9, 10]. In summary, the downregulation of LXRβ and BLBP observed in this study may impair cortical development by disrupting RGCs' function and astrocyte differentiation.

This study examined the regulatory effects of the LXRβ agonist TO901317 and the LXRβ antagonist GSK2033 on glial migration, differentiation, and cortical function. Previous research has shown that LXRβ agonists have significant anti‐inflammatory and neuroprotective effects in various neurodegenerative disease models (such as Alzheimer's disease, Parkinson's disease, and stroke) [22, 23, 24]. LXRβ activation inhibits the NF‐κB pathway and reduces the M1 polarization of microglia, thereby alleviating neuroinflammation [25]. In addition, LXRβ activation promotes neuronal survival and neural progenitor cell proliferation through the PI3K/Akt pathway, thereby improving cognitive dysfunction [26]. In our FL‐CD model, LXRβ agonists significantly reduced the cortical glial proliferation and effectively regulated glial migration patterns. Immunofluorescence analysis showed that while astrocyte activation was observed across all experimental groups, the group treated with LXRβ agonists exhibited less astrocyte migration and proliferation, with a reduced glial migration flow. The attenuation of glial migration observed after TO901317 treatment may stem from LXRβ‐dependent regulation of lipid metabolism. LXRβ activation upregulates ApoE and the cholesterol efflux transporters ABCA1 and ABCG1, which alters membrane lipid‐raft organization and thereby affects downstream signaling pathways governing glial motility [13, 27]. This phenomenon is consistent with the fact that LXRβ agonists inhibit neuroinflammation and reduce the production of reactive astrocytes [28]. In addition to its general anti‐inflammatory effects, LXRβ activation can trans‐repress NF‐κB signaling and reduce the expression of chemokines such as MCP‐1/CCL2, thereby flattening the chemoattractant gradients that normally drive directed glial streaming toward the lesion [29]. Quantitative analysis further revealed that in the LXRβ agonist group, GFAP‐positive cells gradually decreased over time while LXRβ/GFAP double‐labeled cells significantly increased, indicating that LXRβ activation may reduce neuroinflammatory responses by regulating the homeostasis of glial cells. In contrast, the LXRβ antagonist group exhibited heightened neuroinflammation. Immunohistochemistry and immunofluorescence analysis showed that the proliferation of astrocytes in the antagonist‐treated group increased significantly, while the number of LXRβ/GFAP double‐labeled positive cells decreased significantly, suggesting that LXRβ antagonism may lead to the destruction of glial homeostasis, thereby exacerbating neuroinflammation. In particular, at P7 and P10, the FL‐CD + GSK group showed more significant glial proliferation, further supporting the importance of LXRβ in maintaining glial function and neural homeostasis [30, 31]. At the molecular level, LXRβ appears to influence not only inflammatory reactivity but also the differentiation and migration programs that shape the glial migration stream. In addition, the number and proportion of BLBP/GFAP double‐labeled positive cells also provided us with valuable clues about the glial migration process. We observed that in the FL‐CD + GSK group, the proportion of BLBP/GFAP double‐labeled positive cells increased significantly, suggesting excessive migration and transformation of glial cells after lesion. In the FL‐CD + TO group, the proportion of BLBP/GFAP double‐labeled positive cells decreased significantly. The observed alteration in BLBP/GFAP co‐expression may be partly explained by indirect regulation of the BLBP pathway. BLBP, a hallmark of radial‐glia‐like reactive astrocytes, is transcriptionally controlled by REV‐ERBα and RORα; as an RXR heterodimer partner, LXRβ may intersect this nuclear‐receptor network and, by lowering inflammatory tone, secondarily suppress BLBP‐linked migratory activity [32]. These results also confirmed from another perspective that activation of LXRβ may inhibit the excessive migration and transformation of glial cells, thereby reducing glial reactions and playing a protective role in the central nervous system. Furthermore, developmental studies have demonstrated that loss of LXRβ disrupts cortical lamination and radial glial scaffolding, underscoring the close relationship between LXRβ signaling and cellular migration programs in the developing cortex [14]. Although previous studies have focused on the anti‐inflammatory and neuroprotective roles of LXRβ agonists in neurodegenerative diseases, this study is the first to investigate their application in an FCD‐related animal model and their direct effects on glial migration and differentiation. Interestingly, the regulation of LXRβ mainly affects the differentiation process of RGCs into astrocytes, rather than directly affecting the activation state of cells. Compared to previous findings that LXRβ activation reduces microglial activation [28], our study further reveals the differences between LXRβ agonists and antagonists in regulating glial differentiation, providing a new perspective for understanding the pathogenesis of FCD.

Expanding on these findings, the LXRβ agonist group exhibited a significant increase in α‐band relative power on the lesion side and a marked decrease in β‐band relative power. Moreover, the *θ*/*β* ratio and functional connectivity analyses also showed enhanced high‐frequency (gamma band) integration ability. These findings suggest that the activation of LXRβ may promote the remodeling of certain key neural networks after cortical lesion. Recent studies have shown that γ‐band synchronization critically depends on inhibitory interneuron activity. Parvalbumin (PV)–expressing interneurons coordinate interhemispheric γ coupling through callosal GABAergic projections, and disruption of these pathways weakens γ coherence and cognitive flexibility, indicating that stronger inhibitory network function supports γ‐band connectivity [33]. On the one hand, several previous studies on FCD have pointed out that high‐frequency oscillations and γ‐band connections are often used to assess epileptogenic zone activation and are closely related to abnormal local neuronal discharges or lesion activity [34, 35, 36, 37, 38]; on the other hand, when using graph theory analysis and EEG/MEG functional connectivity evaluation, FCD lesions often show enhanced connectivity in the β and γ bands and increased network efficiency [36, 39], which echoes the results of enhanced coherence in the γ bands of channels 2–3 and channels 3–4 observed in the agonist group in this study.

At the circuit level, PV‐mediated GABAergic transmission is essential for maintaining synchronous γ activity; its downregulation or NMDA receptor dysfunction reduces γ amplitude and network coordination [40, 41]. These observations provide a physiological basis for interpreting the enhanced γ connectivity in the LXRβ‐agonist group as reflecting improved inhibitory network coordination. At the cellular and molecular levels, accumulating evidence highlights the critical role in regulating the excitability of neural circuits and the pathological process of epilepsy [42, 43, 44, 45, 46]. Dysfunction in astrocytic glutamate clearance, potassium ion redistribution or neuroactive molecule release can lead to local neuronal depolarization and abnormal synchronous discharge [47, 48, 49]. Besides, LXR agonists have been shown to affect synaptic transmission and plasticity of neural circuits, especially reducing the frequency and amplitude of spontaneous inhibitory postsynaptic currents in areas such as the striatum [50]. Moreover, maturation of PV interneuron networks and GABA_a_‐receptor–mediated inhibition is known to stabilize γ oscillations and strengthen γ‐band connectivity [51, 52, 53]. Together, these findings suggest that the γ‐band enhancement observed in this study may arise from LXRβ‐dependent modulation of inhibitory network synchrony. Based on these findings, we propose that after cortical lesion, LXRβ activation stabilizes the astrocytic microenvironment (e.g., ion buffering, glutamate clearance and inflammation regulation) and enhances inhibitory network function. This reduces high‐amplitude β‐band discharges, promotes α rhythm organization and strengthens γ‐band connectivity while preventing excessive synchronization. In contrast, inhibiting LXRβ disrupts astrocytic homeostasis, leading to excessive glial proliferation and abnormal excitability. As a result, this leads to persistent high‐frequency discharges, β‐band abnormalities and reduced neural plasticity, making it harder to restore normal rhythms. Conversely, the changes in the above electrophysiological indicators (such as α/β ratio and γ‐band connectivity) of LXRβ inhibitors were not as significant as those of the agonist group, suggesting that LXRβ and its related downstream pathways may play a relatively key driving role in the remodeling of brain excitability. To date, no studies have directly investigated the effects of LXRβ on cortical EEG activity; our findings may help bridge this gap. In summary, the observed changes in EEG frequency band power and network functional connectivity in this study are likely to be partly derived from the regulation of LXRβ on astrocyte‐neuron circuits; it not only reflects the reconstruction of the excitation‐inhibition balance of local regions after damage, but also shows certain similarities with the mechanism of high‐frequency band activity and abnormal discharge in disease models such as FCD. Future studies should further elucidate the precise relationship between the LXRβ signaling pathway and brain network remodeling by tracking astrocyte activation states and presynaptic/postsynaptic ion flow dynamics.

Although this study sheds light on key mechanisms, it has some limitations. First, while the FL‐CD model effectively replicates key pathological features of FCD, such as cortical heterotopia, microgyria and abnormal neuronal arrangement, it is still different from the complex and diverse pathogenesis of human FCD, particularly regarding lesion progression and anatomical distribution. As a result, its correspondence to clinical cases remains incomplete. Second, this study primarily focuses on the critical developmental window from early to sub‐mature stages, lacking long‐term microscopic and macroscopic observations into adulthood or aging. This limitation makes it difficult to fully assess the prolonged evolution of FCD lesions over time. Third, although LXRβ agonists and antagonists were core intervention methods to our investigation of glial and cortical function regulation, FCD involves multiple molecular pathways, including mTOR, TSC1/2, PI3K/Akt and so on. LXRβ represents only one regulatory node; the potential synergistic or antagonistic effects of other signals still need to be further explored. To address these limitations, observations will be extended in future research, utilizing LXRβ knock‐out models and integrating continuous EEG monitoring with multidimensional behavioral assessments. Besides, some histological findings in this study were qualitative; future stereological analyses will quantitatively verify these spatial patterns. These approaches will enable a more comprehensive evaluation of the long‐term effects of early interventions, ultimately guiding more effective strategies for the early comprehensive prevention and treatment of FCD.

## 5 Conclusion

In summary, based on the FL‐CD model, this study highlights the critical regulatory role of LXRβ in FCD pathogenesis, particularly in astrocyte proliferation, differentiation and cortical network remodeling. By pharmacologically modulating LXRβ, we observed its essential role in maintaining the homeostasis of the glial‐neuronal microenvironment and regulating electrophysiological activities. These findings offer new insights into FCD pathogenesis and establish a foundation for future LXRβ‐targeted interventions, further expanding its translational potential in FCD and related neurodevelopmental disorders.

## Author Contributions

Zhi Zhang, Di Du and Min Song were responsible for the establishment and management of animal models. Jie Li and Xinning Dong were in charge of the western blot experiment. Yiwen Mei, Zhi Zhang and Jinwei Zhang were responsible for the immunohistochemical experiment. Xin Chen and Zhi Zhang were responsible for the electroencephalogram experiments. Xin Chen, Ming Zhang and Sixun Yu were responsible for data analysis. Xin Chen, Haifeng Shu and Yuan Ma were responsible for writing the manuscripts.

## Funding

This work was supported by Joint key project [grant numbers 2019LH01]; Department of Science and Technology of Sichuan Province [grant numbers 22CXRC0178]; Medical Innovation Project [grant numbers 21WQ040]; Hospital Management Project of Western Command General Hospital [grant numbers 2021‐XZYG‐B22]; Hospital Management Project of Western Command General Hospital [grant numbers 2021‐XZYG‐B21].

## Ethics Statement

All animal experiments were approved by the Animal Care and Use Committee of the General Hospital of the Western Theater Command under the approval ID NSFCKY137‐1. All procedures complied with national and international guidelines for the care and use of laboratory animals.

## Consent

Every author approved the manuscript before submission for publication.

## Conflicts of Interest

The authors declare no conflicts of interest.

## Supporting information


**Figure S1:** Distribution of LXRβ/GFAP and BLBP/GFAP co‐labeled cells in the lesion cortex at the early stage (P2) of drug intervention in FL‐CD model.

**Figure S2:** Distribution of LXRβ/GFAP co‐labeled cells in the “glial migration flow” at P7 after drug intervention in FL‐CD model.

**Figure S3:** Co‐localization of LXRβ and BLBP with neuronal and microglial markers in the lesion area.

## Data Availability

The data that support the findings of this study are available from the corresponding author upon reasonable request.
