# Supplementary figures and images for: Liver X Receptor Beta Regulates Glial Dynamics and Cortical Network Remodeling in a Freezing Lesion–Cortical Dysplasia Model
Source: CNS Neurosci Ther. 2025 Nov 26;31(11):e70671. doi: 10.1111/cns.70671 (PMC12657261; doi:10.1111/cns.70671)

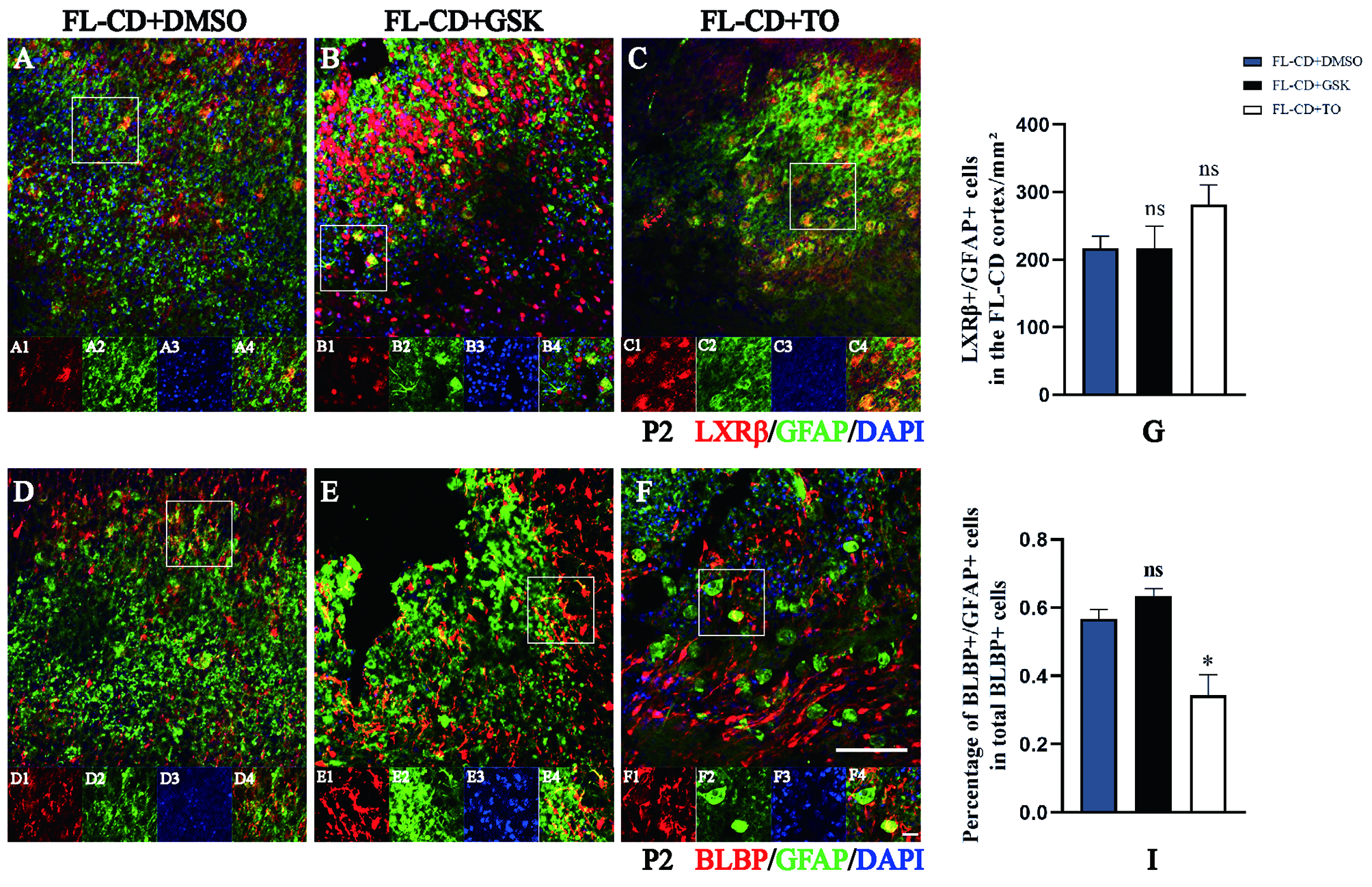

Supplement: Supplementary file 1 — Figure S1: Distribution of LXRβ/GFAP and BLBP/GFAP co‐labeled cells in the lesion cortex at the early stage (P2) of drug intervention in FL‐CD model. [file CNS-31-e70671-s003.tif]

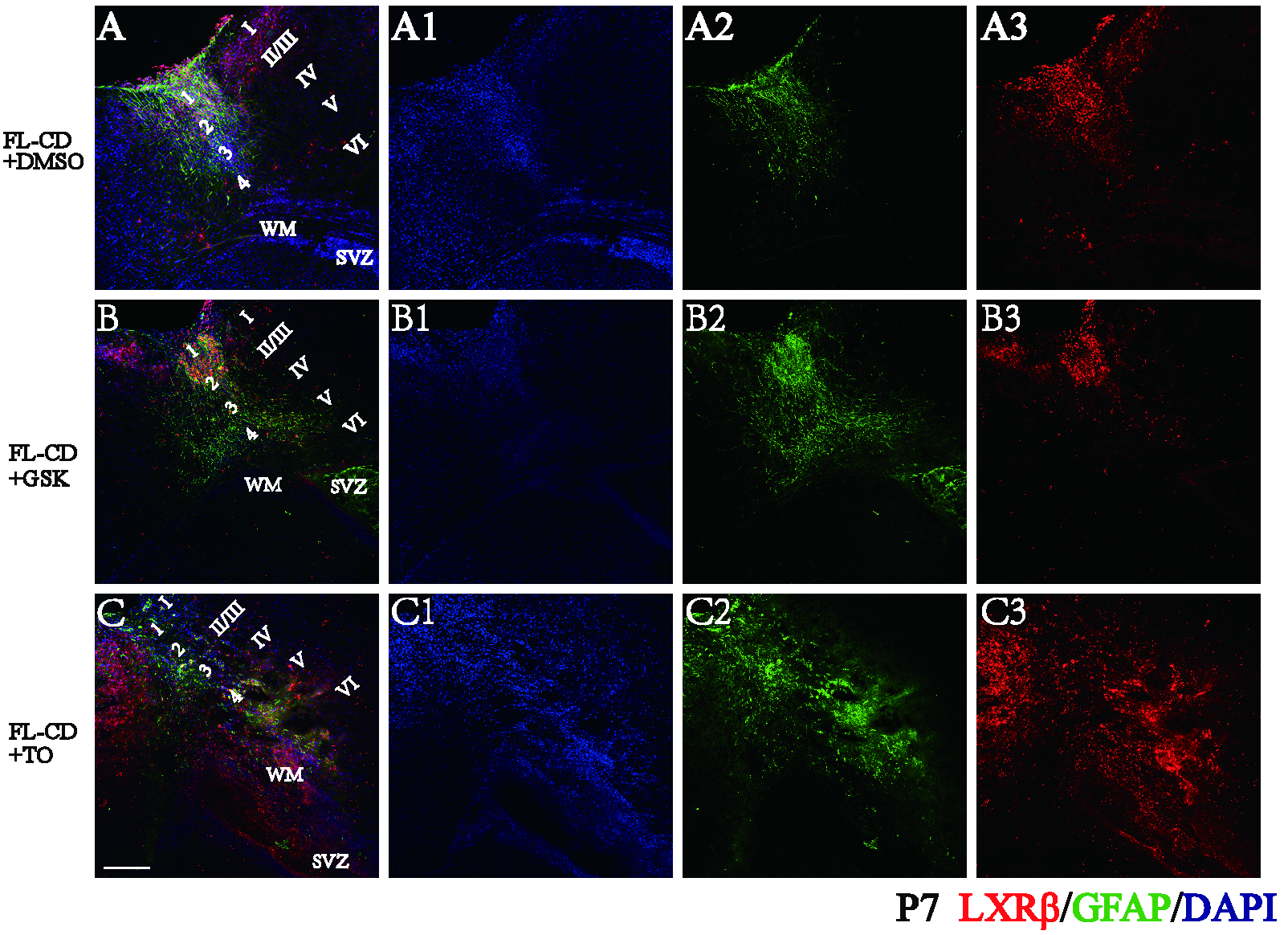

Supplement: Supplementary file 2 — Figure S2: Distribution of LXRβ/GFAP co‐labeled cells in the “glial migration flow” at P7 after drug intervention in FL‐CD model. [file CNS-31-e70671-s002.tif]

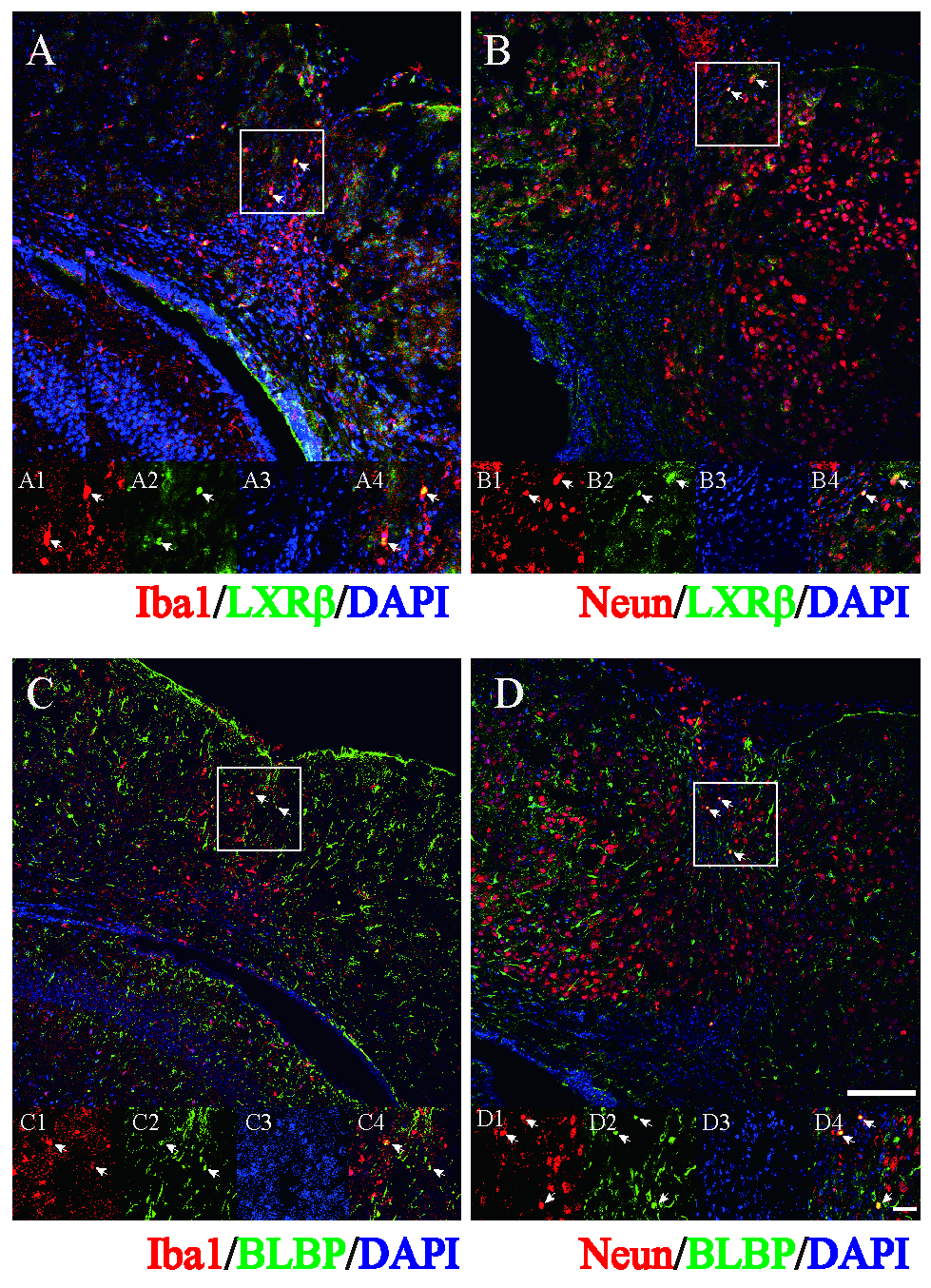

Supplement: Supplementary file 3 — Figure S3: Co‐localization of LXRβ and BLBP with neuronal and microglial markers in the lesion area. [file CNS-31-e70671-s001.tif]
